# Identification of Recombinant Chimpanzee Adenovirus C68 Degradation Products Detected by AEX-HPLC

**DOI:** 10.3389/fbioe.2022.753481

**Published:** 2022-04-05

**Authors:** Thomas W. Powers, Elise K. Mullins, Kun Zhang, Joseph J. Binder, Olga Friese, Herbert A. Runnels, Lawrence C. Thompson

**Affiliations:** ^1^ Analytical Research and Development, Biotherapeutic Pharmaceutical Sciences, Pfizer Inc., Chesterfield, MO, United States; ^2^ Cancer Vaccines and Immunotherapeutics, Pfizer Inc., San Diego, CA, United States

**Keywords:** adenovirus, AEX-HPLC, deamidation, hexon, mass spectometry, kinetics, non-human primate

## Abstract

Physicochemical tests represent important tools for the analytical control strategy of biotherapeutics. For adenoviral modalities, anion-exchange high performance liquid chromatography (AEX-HPLC) represents an important methodology, as it is able to simultaneously provide information on viral particle concentration, product purity and surface charge in a high-throughput manner. During product development of an adenoviral-based therapeutic, an accelerated stability study was performed and showed changes in each of the AEX-HPLC reportable attributes. These changes also correlated with a decrease in product infectivity prompting a detailed characterization of the impurity and mechanism of the surface charge change. Characterization experiments identified the impurity to be free hexon trimer, suggesting that capsid degradation could be contributing to both the impurity and reduced particle concentration. Additional mass spectrometry characterization identified deamidation of specific hexon residues to be associated with the external surface charge modification observed upon thermal stress conditions. To demonstrate a causal relationship between deamidation and surface charge changes observed by AEX-HPLC, site-directed mutagenesis experiments were performed. Through this effort, it was concluded that deamidation of asparagine 414 was responsible for the surface charge alteration observed in the AEX-HPLC profile but was not associated with the reduction in infectivity. Overall, this manuscript details critical characterization efforts conducted to enable understanding of a pivotal physicochemical test for adenoviral based therapeutics.

## Introduction

Non-human primate adenoviral vectors are attractive for use in human vaccine and immuno-oncological applications as they maintain many of the attributes that make adenovirus appealing as a therapeutic vector while having reduced host pre-existing antivector immunity (Capone et al., 2013; Cheng et al., 2015; Zhang et al., 2017; Zhao et al., 2018; Mullins et al., 2021). Recent successes using these constructs include modified chimpanzee adenovirus type 3–vectored ebolavirus vaccine (cAd3-EBO) (Stanley et al., 2014; Ledgerwood et al., 2017) and replication-deficient simian adenoviral vectored vaccine ChAdOx1 nCoV-19 against SARS-CoV-2 (Folegatti et al., 2020; Ewer et al., 2021; Putter, 2021).

As with any therapeutic product, the analytical control strategy exists for the purpose of ensuring product safety and efficacy of these vectors. The analytical control strategy must include methods that demonstrate product quality throughout manufacturing, shipping, and storage. Cell-based bioassays are an important component of the analytical control strategy, as they provide *In vitro* measurements of critical quality attributes like infectivity, protein expression/function, etc. (Ich, 1999; FDA, 2011; CHMP, 2019; FDA, 2020). While these assays are vital to evaluate overall potency, everyday use of these bioassays is resource and reagent intensive. Therefore, it is desirable to have higher throughput physicochemical techniques as a part of the analytical control strategy that give an analogous assessment of product quality but can be run routinely during process development, formulation development, and on fully purified products. For adenoviruses, these options vary but include many well-known techniques such as AEX-HPLC (Blanche et al., 2000; Klyushnichenko et al., 2001; Kuhn et al., 2007; Whitfield et al., 2009; Kramberger et al., 2015), RP-HPLC (Lehmberg et al., 1999; Blanche et al., 2001; Vellekamp et al., 2001; Chelius et al., 2002; Takahashi et al., 2006; Benevento et al., 2014; Pérez-Berná et al., 2014; van Tricht et al., 2018), capillary electrophoresis (CE) (van Tricht et al., 2017), analytical ultracentrifugation (AUC) (Berkowitz and Philo, 2007; Berkowitz, 2008; Yang et al., 2008) and differential centrifugal sedimentation (DCS) (Bondoc and Fitzpatrick, 1998; Shih et al., 2010). AEX-HPLC is the most widely applied of these for in-process, release and stability testing, as AEX-HPLC can simultaneously determine viral particle concentration, product purity and surface charge.

A subset of the methods in the analytical control strategy should be capable of detecting product deterioration over time, which classifies the methods as stability-indicating assays. Performing accelerated stability studies, in which materials are subjected to elevated temperatures compared to recommended storage conditions, enables a more rapid product degradation and is often used to determine which methods are stability indicating, including both physiochemical assays and bioassays. Correlating the changes between physiochemical assays and bioassays provides information about which quality attributes may play important functional roles. In the current study, two chimpanzee adenovirus C68 constructs (heretofore known as AdC68) (Farina et al., 2001; Cohen et al., 2002; Xiang et al., 2002; Tatsis et al., 2006) were subjected to an accelerated stability study and the resulting materials were characterized with analytical assays in place to support the overall control strategy. The data revealed several changes in the AEX-HPLC profile, which correlated with overall decreases in product infectivity. Specifically, AEX-HPLC results demonstrated changes in viral particle concentration, product purity and surface charge. While these results demonstrated that AEX-HPLC was a powerful stability indicating tool, the method is unable to characterize specific attributes that change on the adenovirus quaternary structure and/or the underlying adenovirus DNA and protein components.

In an effort to better understand the attributes that changed in the AEX-HPLC data, and to further understand if these attributes were responsible for the observed decrease in infectivity, we present a characterization strategy for the identification of adenovirus molecular modifications and product related impurities. This strategy utilizes the power of mass spectrometry to identify changes in quality attributes at the molecular level and reveal degradation hotspots. The implementation of these approaches was supplemented by analytical fraction collection, enabling a targeted assessment of AEX-HPLC peaks. As chemical modifications were identified, the locations of the attributes were modeled in-silico. Furthermore, site-specific mutagenesis was performed to mimic the modifications and assess the impact of the attributes both on the AEX-HPLC assay and on the product infectivity. The study documented herein provides an in-depth evaluation of attributes and modifications that are responsible for changes in the AEX-HPLC assay and enhances knowledge around quality attributes for adenoviral vectors.

## Materials and Methods

### Materials and Formulation

Two AdC68 constructs were manufactured within Pfizer. AdC68 material was formulated in A195 buffer (Xiang et al., 2002): 10 mM Tris, 75 mM NaCl, 5% w/v sucrose, 0.02% (w/v) 80 polysorbate 80, 1.0mM MgCl2, 0.1 mM EDTA, 0.5% (v/v) ethanol, 10 mM L-histidine, pH 7.4 (Mullins et al., 2021).

### Accelerate Stability of Adenovirus Material

An accelerated stability study was performed in which adenovirus material was incubated at 26.5°C for up to 12 weeks. Throughout product development, these conditions were demonstrated to yield significant product degradation. A bulk AdC68 material was incubated at 26.5°C, and incremental time points were pulled from the bulk material and frozen at -80°C prior to analysis.

### Determination of the Adenoviral Infectivity by a Cell-Based Adenovirus Titer Immunoassay

An adenoviral infectivity method was developed to monitor infectivity. For this assay, cultures of HEK293 cells (Cell biolabs, Catalog No. AD-100) were grown in a flask containing Dulbecco’s modified medium (Gibco, Catalog No. 11995) supplemented with 10% Heat Inactivated Fetal Bovine Serum (Gibco, Catalog No. 10082) and 1x Penicillin-Streptomycin (Gibco, Catalog No. 15140) in a 5% CO_2_ atmosphere at 37°C. HEK293 cells were seeded into a 24-well flat bottom cell culture plate at 2.2E5 cells/well and then incubated in a 37°C, 5% CO_2_ incubator. A dilution series of adenovirus sample was prepared in cell culture medium. The dilution series was then added to the wells of the cell assay plate about 1 h after cell seeding. The assay plate was then incubated for 2 days. The cells were then fixed with cold methanol at -20°C and then stained with a polyclonal antibody against adenovirus Hexon proteins, which is expressed upon infection of the cells with adenovirus sample. Positive stained cells in assay wells were counted under microscope fitted with a ×10 lens. Cells per view field were recorded and used to calculate the adenovirus sample infectivity, reported as infectious units (IFU)/mL as well as viral particles (vp)/IFU.

### AEX-HPLC

#### Analytical AEX-HPLC

The analytical AEX-HPLC method was used to determine product purity, virus particle concentration, and surface charge through the measurement of relative retention time. The analytical AEX-HPLC method used a high affinity, anion exchange column (GE Healthcare, Resource Q, 1 ml 15 μm, 6.4 × 30 mm, Part No. 17-1177-01) to separate the AdC68 and impurities. AdC68 samples were diluted with formulation buffer and analyzed against a standard curve that ran from 0.25E11 VP/mL to 3.0E11 VP/mL. Elution of the bound molecules was achieved using a salt gradient with a flow rate of 1.0 ml/min over the course of 30 min. The mobile phases consisted of 50 mM Tris, 0.05% Tween 80, 0.1 mM EDTA pH 8.0, with the addition of 1 M NaCl to mobile phase B. The gradient ranged from 10 to 100 %B. The AEX-HPLC was coupled to a fluorescence detector which was set to collect data with an excitation of 280 nm and emission of 320 nm.

Data were processed to monitor peak areas and relative retention times. Relative purity was determined by comparing area counts of the eluting peaks and was reported as a percent of the total area for all peaks. Particle concentration was achieved by correlation of the unknowns to a standard curve. Relative retention time (acidic shift) were measured by comparing the elution time of the sample to the averaged elution time of the standard curve samples. The method was qualified and determined to be adequately precise for particle concentration, purity and relative retention time.

#### Preparative AEX-HPLC Method

For in-depth characterization, fraction collection was utilized to isolate the impurity peak from the main peak in the AEX-HPLC profile. A CIMac Adeno 0.1 ml analytical column (Sartorius Part #110.8502-2) was set up on an Akta Explorer FPLC system. This column was utilized in order to achieve complete baseline separation of the impurity peak and main peak in a preparative scale method. An increasing salt gradient within a Tris buffer system was applied to elute the adenovirus fractions from the column. The mobile phases consisted of 50 mM Tris, pH 7.5 with the addition of 2 M NaCl to mobile phase B. The gradient ranged from 7.5 to 18 %B. Fractions were collected from bulk adenovirus material that had previously been degraded at 26.5°C for 12 weeks. For the fraction collection experiments, a series of 500 µL injections were made across the column with several fractions taken throughout the run. The column was flushed and then this was repeated several times to yield enough fractionated material for additional experiments.

### Characterization of Adenoviral Capsid by Mass Spectrometry

#### Characterization of Intact Capsid Proteins by RP-HPLC/MS

The denatured capsid proteins were chromatographically separated on a Phenomenex Jupiter C4, 5 µm, 2 × 150 mm, 300Å analytical column (Part #00F-4167-B0) using an Agilent 1200 HPLC system similar to what was described previously (Vellekamp et al., 2001; Mullins et al., 2021) with the following alterations. The column was held at a temperature of 5 0°C with a gradient consisting of 20–100% acetonitrile with trifluoroacetic acid (TFA) over 90 min with a flow rate of 0.2 ml/min. Prior to analysis, samples were denatured with acetonitrile and TFA to yield a final mixture of 20% ACN, 0.1% TFA. For each sample, 4.4E10 viral particles of adenovirus were loaded onto the column.

Intact mass spectra were acquired in two ways. The Agilent 1200 HPLC was coupled to a Thermo Orbitrap EMR mass spectrometer. Samples were analyzed in positive-ion mode with a detection range of 400–6,000 m/z. Data were collected with a resolution of 17,500 and an isCID of 50 eV. Additionally, a Bruker maXis II QTOF mass spectrometer connected to a Waters HClass UHPLC was utilized. Samples were analyzed in positive-ion mode with a detection range of 400–4,000 m*/z*. The instrument was calibrated with the Agilent Tune Mix for ESI-QTOF MS instruments. The capillary voltage was 4,000, isCID was 150 eV, collision energy was 15 eV, and the transfer time was set to 130 µs? Thermo data were processed using PMI (Protein Metrics) Intact deconvolution software and Bruker data were processed with Bruker DataAnalysis software.

#### Characterization of Intact Capsid Proteins by Native SEC-HPLC/MS

Fractions collected by AEX-HPLC were analyzed by native SEC-HPLC/MS. AEX-HPLC fractions were collected as described in *Preparative AEX-HPLC Method* Section. The impurity fraction was injected onto a ACQUITY UPLC Protein BEH SEC Column 200Å, 1.7 µm, 4.6 × 300 mm column (Part #186005225) using an Agilent 1200 HPLC system. The chromatography consisted of an isocratic gradient with 40 mM ammonium formate at a constant 25°C column temperature. The HPLC was connected to an Thermo Orbitrap EMR mass spectrometer with a resolution of 17,500 and an isCID of 85 eV. Data were processed using PMI (Protein Metrics) Intact deconvolution software.

#### Characterization of Adenoviral Peptides by RP-HPLC/MS/MS

AdC68 was denatured and digested similar to what was described previously for human Ad5 (Benevento et al., 2014) with the following alterations. Prior to digestion, six vials of AdC68 reference material were pooled together, diluted to a total volume of 10 ml with formulation buffer, and concentrated in an Ultra-15 10 kD MWCO centrifugal filter (M012160) at 3,300 g for 20 min. The retentate was removed ( ∼500 µL) and denatured with 500 µL of 50 mM Tris pH 8.0 with 10% sodium deoxycholate at 95°C for 5 min. The sample was then reduced with 10 µL of 1 M DTT at 56°C for 5 minutes and alkylated with 20 µL of 1 M IAA at 37°C for 15 min in the dark. The sample was mixed with 9 ml of 50 mM Tris pH 8.0 with 0.02% sodium deoxycholate and an additional buffer exchange was performed in an Ultra-15 10 kD MWCO centrifugal filter at 3,300 g for 20 min. An additional 10 ml of 50 mM Tris pH 8.0 with 0.02% sodium deoxycholate was added to the sample and centrifugation was repeated to remove excess sodium deoxycholate. The retentate were then removed and the filter was washed with 50 mM Tris pH 8.0 with 0.02% sodium deoxycholate to yield a final sample volume of 500 µL. The sample was digested with 20 µg trypsin for 15 min at 37°C followed by DNase1 digestion (20U) for 15 min at 37°C. Enzymatic digestion was quenched by the addition of 50 µL of 10% TFA and precipitate was removed by centrifugation at 20,000 g for 2 min. The supernatant was removed and added to an HPLC vial for analysis.

Fractions from the preparative AEX-HPLC method were digested using the approach outlined above. Fractions were concentrated to 500 µL prior to denaturation with 500 µL of 50 mM Tris pH 8.0 with 10% sodium deoxycholate at 95°C for 5 min. Reduction, alkylation, buffer exchange and digestion followed the experimental procedure described for the unfractionated adenovirus material. Proteolytic digested material was chromatographically separated on an Acquity UPLC BEH C18, 2.1 × 300 mm, 1.7 µm, 130Å analytical column (Part #186005792) using an Agilent 1260 HPLC. The column was held at a temperature of 60°C with a gradient consisting of 1–98% acetonitrile with trifluoroacetic acid over 315 min with a flow rate of 0.2 ml/min.

The Agilent 1260 HPLC was coupled to a Thermo Q-Exactive Plus Orbitrap mass spectrometer. Samples were analyzed in positive-ion mode with a detection range of 300–1650 m/z. The instrument utilized an internal lock mass ion of hexakis (1H,1H,3H-perfluoropropoxy)-phosphazene at m/z 922.009798 for [M + H+]1 + for dynamic calibration. Full-scan MS spectra were acquired in the Orbitrap with a resolution of 70,000 at m/z 400. The ten most intense ions at a threshold above 2,700 were selected for collision-induced fragmentation and analyzed in the orbitrap at a normalized collision energy of 30 and an isolation width of 3.0 m/z.

The data were processed with BioPharma Finder (v3.0, ThermoFisher Scientific) and searched against a library of AdC68 protein sequences. Variable modifications, including deamidation, oxidation, NH3 loss, water loss and acetylation were included. Relative quantitation was computed in BioPharma Finder. All experiments were run in single injections, so the relative abundances reported herein represent data from one instance.

### Mutagenesis

Select site-specific mutagenesis to the AdC68 construct was performed to investigate the impact of capsid deamidation. The mutation to the construct was performed by the company SGI-DNA. Mutations of N76, N414 and N76 & N414 on the hexon protein were generated, converting the DNA to encode aspartic acid from the original asparagine. Materials were manufactured and purified by Viraquest Inc., North Liberty, IA.

## Results

### AEX-HPLC is a Stability-Indicating Assay

Multiple recombinant AdC68 constructs were designed during development with two selected to move forward into clinical trials (heretofore named AdC68 #1 and #2). In preparation for clinical manufacturing, infectivity and an AEX-HPLC purity assays (see Materials and Methods) were developed and qualified as part of the overall analytical control strategy. These tests were then utilized to evaluate samples from the 12-weeks accelerated stability study at 26.5°C to demonstrate these assays were stability indicating. Tables 1, 2 show that the infectivity of both developmental drug substance constructs decreased with a corresponding loss of purity and viral particle titer. Furthermore, there was a concomitant increase in the relative retention time, indicative of an acidic shift, of the viral particle peak. All changes observed in the data herein were significantly greater than the method precision results obtained during the method qualification (data not shown), indicating real changes in each attribute. A similar observation of an AEX-HPLC acidic shift for human Ad5 has been observed and investigated previously (Blanche et al., 2001).

**TABLE 1:** Infectivity & AEX-HPLC data from the AdC68 #1 accelerated stability time course.

	Infectivity assay	AEX-HPLC			Infectivity per particle load
**Sample**	**IFU/mL**	**% Purity**	**VP/mL**	**RRT**	**VP/IFU**
T0	3.08E9	98.7	2.1E11	1.001	68
1w	1.87E9	95.3	1.7E11	1.009	91
2w	1.47E9	91.8	1.4E11	1.018	95
4w	6.63E8	86.4	1.0E11	1.032	151
8w	2.10E7^a^	80.5	7.0E10	1.048	3326^a^
12w	3.16E6^a^	64.7	4.0E10	1.054	12,671^a^

Abbreviations: IFU, infectivity units; VP, viral particles; RRT, relative retention time.

a Extrapolated, outside the calibrated method range.

**TABLE 2:** Infectivity & AEX-HPLC data from the AdC68 #2 accelerated stability time course.

	Infectivity assay	AEX-HPLC			Infectivity per particle load
**Sample**	**IFU/mL**	**% Purity**	**VP/mL**	**RRT**	**VP/IFU**
T0	2.68E9	96.7	2.0E11	0.998	75
1w	1.54E9	93.4	1.6E11	1.008	104
2w	1.48E9	92.5	1.5E11	1.016	101
4w	5.26E8	90.2	1.3E11	1.030	247
8w	2.08E7^a^	85.9	9.0E10	1.050	4330^a^
12w	2.10E6^a^	81.5	7.0E10	1.058	33,261^a^

Abbreviations: IFU, infectivity units; VP, viral particles; RRT, relative retention time.

a xtrapolated, outside the calibrated method range.

**FIGURE 1:**
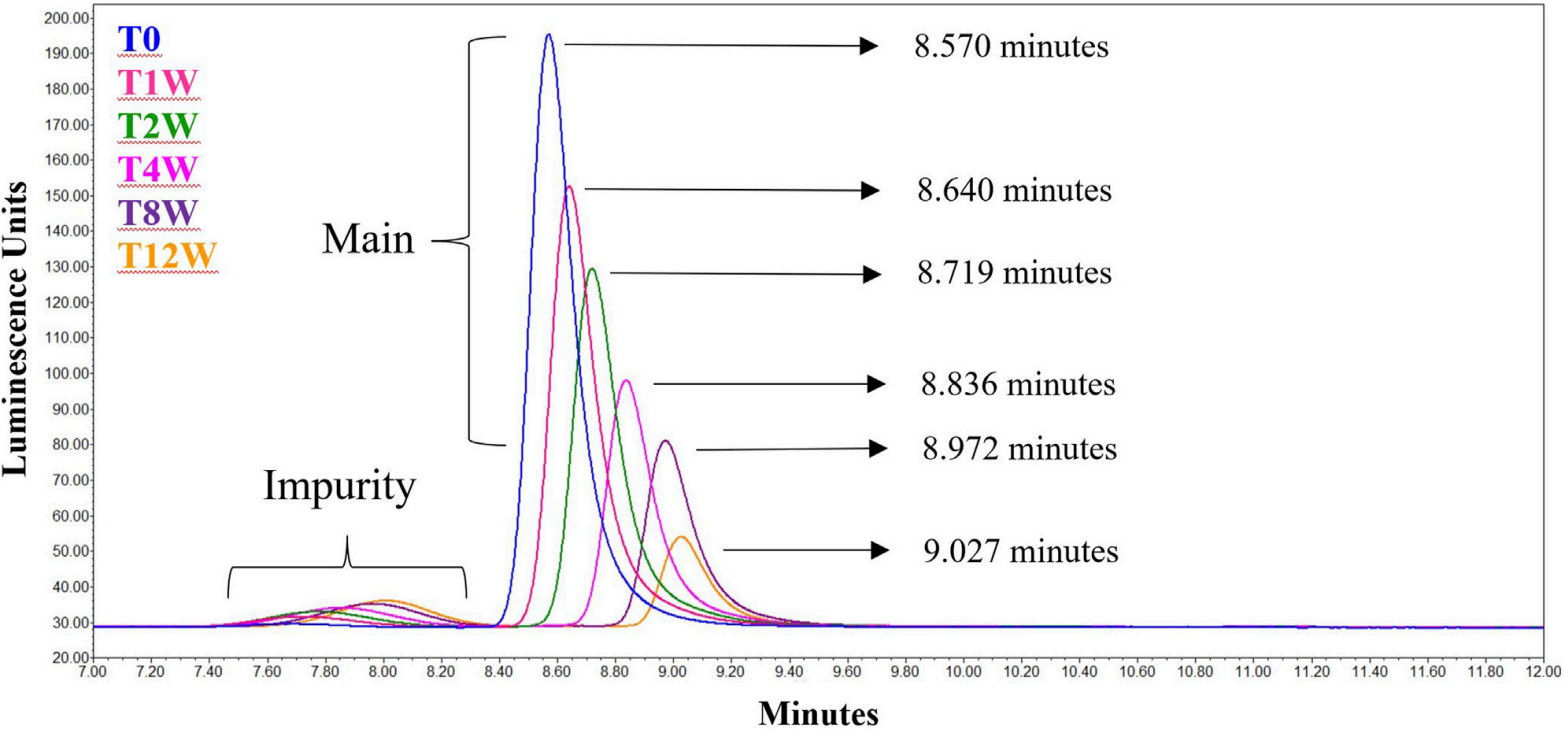
Singlicate AEX-HPLC data from the AdC68 #1 accelerated stability time course. Peaks designated the “Main” and “Impurity” are labeled as such. The timepoints in the accelerated stability study are labeled on the figure, with blue representing the profile of the unstressed material. The retention times for each time point are shown in the figure.

The data from Table 1 can be readily visualized by inspecting overlaid chromatograms (Figure 1) from which the associated AdC68 #1 attribute data was calculated (Table 1). Sequential time points show a drop in the presumed AdC68 (“main”) peak area corresponding to the loss of viral particle titer. The increase in the early eluting “impurity” peak area, along with the reduction in the “main” peak area, corresponds to a decrease in the overall purity. The rightward trajectory of the main AdC68 (“main”) peak across the accelerated stability time course depicts the increase in retention time, which is a surrogate measurement of increased acidic surface charge. While cataloguing the “main” peak as the AdC68 product of interest for viral particle titer determinations (Table 1) was initially an anecdotal identification, additional characterization was used to confirm the “main” and “impurity” peak identities.

### Analysis of AEX Fractions Demonstrates That the Impurity is Hexon Trimer

**FIGURE 2:**
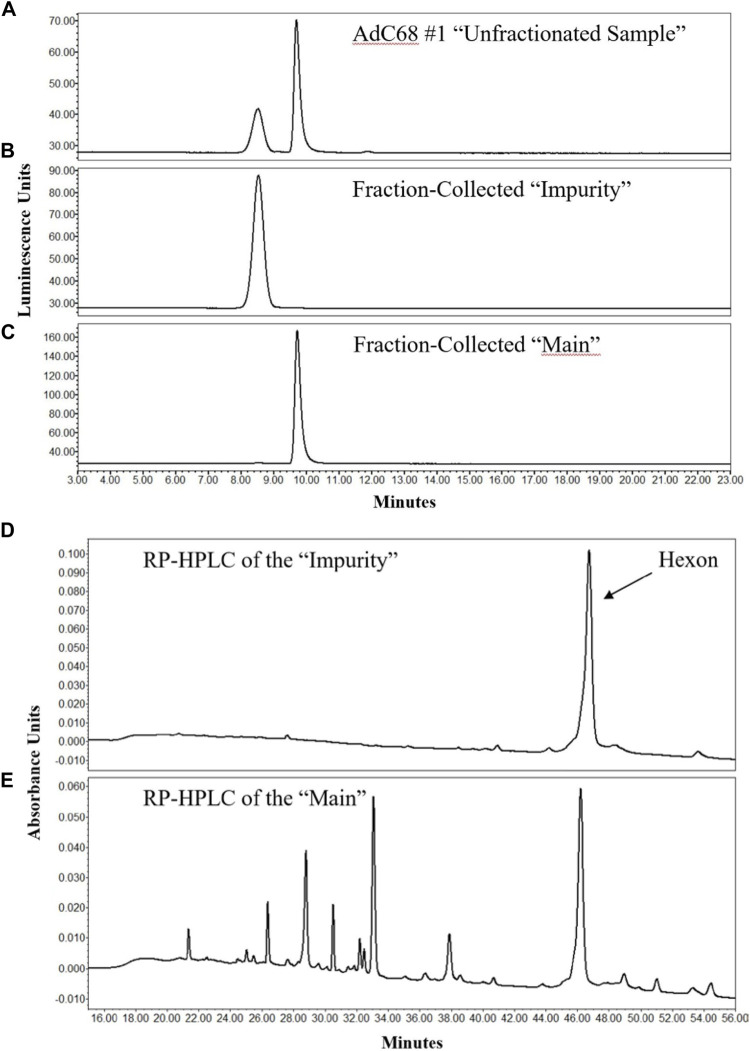
AEX-HPLC and RP-HPLC profiles of the 12-weeks accelerated stability sample. The chromatographic profile of the unfractionated 12-weeks stability sample is shown in **(A)**. After enrichment of the “Impurity” and “Main” peaks by the preparative AEX-HPLC assay, efficient enrichment was demonstrated of the “Impurity” **(B)** and “Main” **(C)** peaks by the analytical AEX-HPLC method. Analyses of fractionated “Impurity” and “Main” peaks by RP-HPLC are shown in **(D,E)**, respectively.

To characterize the “main” and “impurity” peak identities, 12-weeks degraded material was assessed. In Figure 2A, the unfractionated sample was shown to contain ample amounts of both “impurity” and “main” peaks in the analytical AEX-HPLC method. A modified, preparative AEX-HPLC method (see Materials and Methods) was then used to collect fractions of the “main” and “impurity” peaks. After several cycles of fractionation, like fractions were pooled together, concentrated and re-evaluated with the analytical AEX-HPLC method to ensure that the isolated peaks were in fact purified representations of “impurity” and “main” peaks (Figures 2B,C).

These fractions were then evaluated to determine their protein composition. Using a RP-HPLC/MS method similar to that described previously (Takahashi et al., 2006), the individual proteins within the samples were separated from each other for identification. The RP-HPLC/UV data demonstrated that the “impurity” peak contained primarily a single protein (Figure 2D), whereas the “main” peak contained many proteins and was representative of the chromatographic profile expected for adenovirus material (Figure 2E). The proteins from the “impurity” and “main” peaks were further examined by MS, which was directly coupled to the RP-HPLC separation. MS data confirmed that the “impurity” peak was comprised almost entirely of hexon protein, while the “main” peak contained all the proteins expected in a AdC68 viral particle, including hexon (Figures 2D,E) (Rux and Burnett, 2004; Russell, 2009; Nemerow et al., 2012; Ahi and Mittal, 2016). These observations were also consistent with an additional AdC68 construct (Supplementary Figure S1). The masses of hexon from both the main and impurity peaks are provided in Table 3. The observed masses of hexon for both the “main” and “impurity” peaks (104,844.8–104,847.7 Da) were abnormally high, especially when compared to hexon from an unstressed sample (104,842.1 Da), possibly indicative of deamidation of the protein.

**TABLE 3:** Observed Masses for deconvoluted hexon by RP-HPLC/MS and nSEC-HPLC/MS.

Sample	RP-HPLC/MS		nSEC/MS	
	Observed Mass (Da)	Theoretical Mass of monomer (Da)	Observed Mass (Da)	Theoretical Mass of trimer (Da)
AdC68 #1 “Main”	104847.2	104842.0	NT	314526.0
AdC68 #1 “Impurity”	104845.2		314545.2	
AdC68 #2 “Main”	104844.8		NT	
AdC68 #2 “Impurity”	104847.7		314559.7	
AdC68 #2 Unfractionated Non-Stressed Sample	104842.1		NT	

NT, denotes not tested.

RP-HPLC/MS is a denaturing analysis such that secondary, tertiary or quaternary structure of the proteins/complex are not retained. While the RP-HPLC/MS data demonstrated the presence of monomeric hexon, hexon is known to form noncovalent trimers on the adenovirus surface (Pichla-Gollon et al., 2007). To determine if the impurity, now identified as hexon, had such high-order structure, nSEC-HPLC/MS was implemented. The data clearly showed that the non-denatured, fraction collected impurity had a mass consistent with hexon trimer (Table 3). The higher-than-expected mass defect is likely explained by a combination of multiple deamidations, poor desolvation, and challenges with deconvolution at higher m/z.

### HPLC-MS/MS and Directed Mutagenesis Confirms Deamidation is Responsible for the Observed Acidic Shift

**FIGURE 3:**
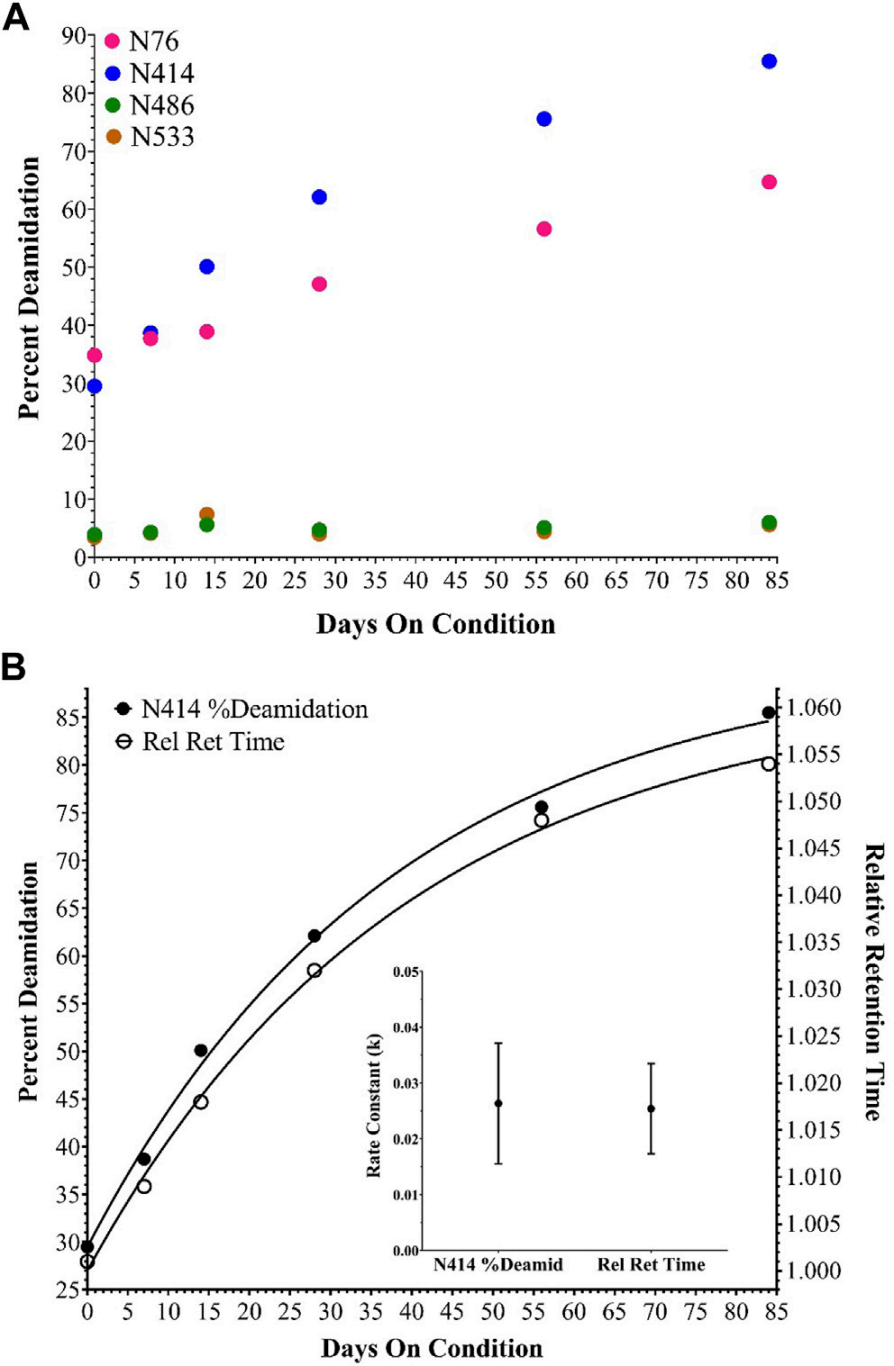
Deamidation of selected hexon asparagine residues during the accelerated stability time course. **(A)** shows percent deamidation determined for N76, 414, 486 and 533 in AdC68 #1. **(B)** shows percent deamidation for N414 and the AEX-HPLC relative retention time shift both fit to a single exponential equation [Y=Y0 + (Plateau-Y0)*(1-exp (-K*x)], the calculated rate constants and the associated error for AdC68 #1.

With the identification of the major impurity concluded, the cause of the acidic shift was investigated by peptide mapping MS experiments. RP-HPLC-MS/MS data from the unfractionated sample time points in the accelerated stability study were collected and searched against various chemical modifications, including deamidation. While several modifications were observed across the entire adenoviral proteome, four hexon deamidation sites were evaluated extensively, including two that changed to a significant degree. Over the time-course, N76 and N414 reached almost 70 and 90% deamidation, respectively (Figure 3A). Conversely, many other sites of deamidation were observed but did not change significantly, including hexon N486 and N533 (Figure 3A).

The relative retention time shift from AEX-HPLC and the percent deamidation of N414 by RP-HPLC-MS/MS were plotted on the same graph and fitted to a single exponential equation to assess the relationship between deamidation and the relative retention time shift. For AdC68 #1, the generated curves and associated rate constants align excellently with each other (Figure 3B). Both curves showed good correlation with a single exponential equation indicating a single kinetic process and both rate constants were within error of matching. These observations were also consistent with an additional AdC68 construct (Supplementary Figure S2).

**FIGURE 4:**
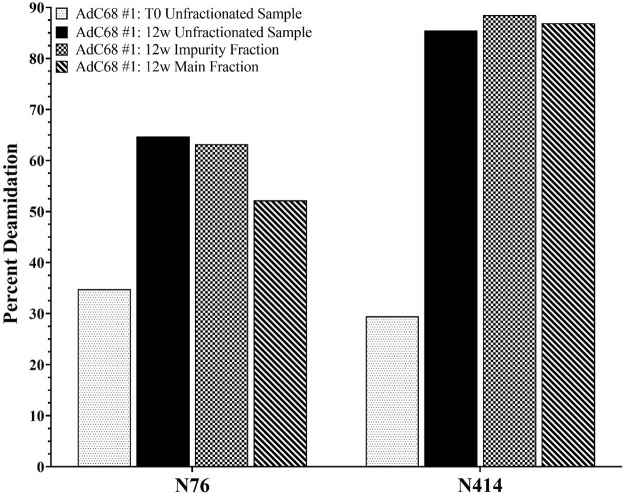
Deamidation level of selected hexon asparagine residues from the 0- and 12-weeks accelerated stability unfractionated samples and the associated “Impurity” and “Main” peaks fractions.

The pooled “impurity” and “main” peaks, along with the unfractionated sample from the 0 and 12-weeks timepoints, were also characterized by RP-HPLC-MS/MS. Specifically, the purpose of this study was to quantitate the deamidation sites of interest established in Figure 3A to ensure that deamidation events were not isolated to the “impurity” peak but also occurred on the intact adenoviral capsid prior to degradation. The data showed similar levels of N414 deamidation across the 12-weeks unfractionated sample and the “main” and “impurity” fractions (Figure 4). The data show somewhat higher levels of N76 deamidation in the “impurity” fraction compared to the “main” fraction (Figure 4). In both cases, the level of deamidation in the “main” fraction and the unfractionated sample at the 12-weeks accelerated timepoint were significantly higher than the 0-weeks timepoint. These observations were also consistent with an additional AdC68 construct (data not shown).

**FIGURE 5:**
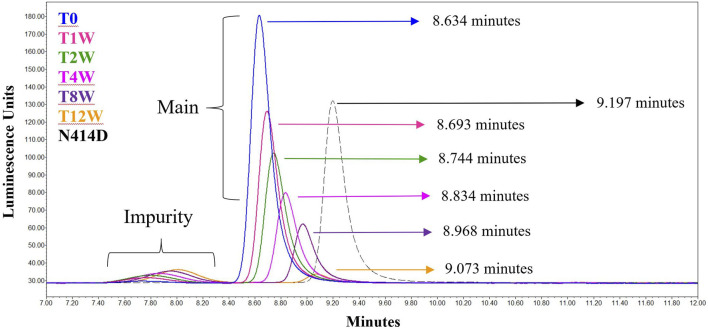
AEX-HPLC of the N414D mutant overlaid with the singlicate AdC68 #1 accelerated stability time course. The timepoints in the accelerated stability study are labeled on the figure, with blue representing the profile of the unstressed material. The gray dashed line represents the N414D mutant. The retention times for each time point and for the mutant are shown in the figure.

To conclude the deamidation inquiry, three hexon mutants were constructed: N414D and N76D single mutants and the N414D/N76D double mutant. These would mimic 100% deamidation at either or both sites for the asparagine residues that showed appreciable increases in deamidation upon accelerated stability. N414D was able to replicate and produce viable, infectious virus (data not shown). To assess the impact of the deamidation mutant on the relative retention time, the N414D mutant was tested by the analytical AEX-HPLC method, alongside the samples from the accelerated stability study (Figure 5). Upon analysis, the modified capsid eluted at a late relative retention time, consistent with the expectation that deamidation causes the AEX-HPLC retention shift in the non-mutant AdC68 accelerated stability samples (Figure 5). In contrast, the N76D single and N414D/N76D double mutant could not produce virus at any appreciable level. As such, the impact to infectivity and relative retention time shift could not be assessed.

## Discussion

As presented in the manuscript, AEX-HPLC represents an important physiochemical method for the adenovirus analytical control strategy. The AEX-HPLC assay is a critical assay for adenovirus-based therapeutics as it is stability indicating (documented herein), higher throughput, and can be reflective of lower-throughput, more resource intensive bioassays. As demonstrated, when the AEX-HPLC assay was used to characterize samples following accelerated stability conditions at 26.5°C, changes in viral particle concentration, particle purity and relative retention time shift were all observed (Table 1; Figure 1). While these changes were observed and correlated with a drop in infectivity, additional work was needed to 1) understand what attributes were driving these changes and 2) assess whether the specific changes were responsible for the reduced infectivity.

### Interpretation of Viral Particle Titer and Particle Purity Results

As observed in Table 1 and Figure 1, viral particle concentration was reduced after accelerated stability conditions, as reflected by the drop in signal in the AEX-HPLC chromatogram. However, based on the magnitude of the particle concentration change *vs*. the magnitude of the decrease in infectivity, the change in particle concentration alone does not account for the decrease in infectivity. This is further demonstrated in the VP/IFU (Table 1) column, which adjusts the infectivity response to the particles dosed. The readout shows substantial reduction in per particle infectivity for the stressed samples.

Similarly, Table 1 and Figure 1 demonstrate a decrease in particle purity after accelerated stability conditions, as reflected by the increase in the “impurity” peak. As with particle concentration, the reduced purity alone would not account for the magnitude of reduced infectivity in the stressed samples. That said, the reduction in particle purity observed by AEX-HPLC does provide an avenue to investigate the mechanism of the viral particle concentration decrease, as the concentration decrease is likely linked to the increase of the impurity.

To understand the identity of the impurity, fractions were collected from the “impurity” and “main” peak observed by the analytical AEX-HPLC method after 12 weeks at 26.5°C. The profile from the unfractionated sample is shown in Figure 2A. A modified, preparative AEX-HPLC method was used for fraction collection to enable more concentrated fractions. Efficient enrichment of the “impurity” and “main” peaks were confirmed with the analytical AEX-HPLC method (Figures 2B,C). After successful enrichment, the “impurity” and “main” fractions were characterized by RP-HPLC/MS to identify differences between the fractions. Interestingly, the “impurity” fraction consisted primarily of a single protein (Figure 2D), which was identified as hexon by RP-HPLC/MS (Table 3). The “main” peak contained a profile indicative of adenovirus with many capsid proteins (Figure 2E), including hexon. RP-HPLC/MS was also used to confirm the identities of the capsid proteins from the “main” peak in an unstressed material, with the mass of hexon shown in Table 3. When compared with a representative unstressed and unfractionated sample, the mass of the “main” and “impurity” fractions from the sample subjected to 12 weeks at 26.5°C had higher observed masses by approximately 2–5 Daltons. This is possibly indicative of several asparagine deamidations, which is frequently observed in accelerated stability studies.

While the “impurity” was identified as hexon, we were interested in whether that “impurity” was monomeric or trimeric hexon, as it is noted that hexon exists as a trimer in the assembled adenovirus capsid (Pichla-Gollon et al., 2007). To assess this, the impurity peaks were evaluated by native SEC-MS, with a gradient that consisted of non-organic mobile phases and gentle MS source parameters directed at keeping non-covalent complexes assembled. The resulting spectra resulted in deconvolved masses between 314,545.2 and 314,559.7 Da, approximately three times the mass of the hexon in the RP-HPLC/MS data, indicating the impurity observed by AEX-HPLC is in fact hexon trimer (Table 3). A comparison of the observed to theoretical mass (314,526.0 Da) represents a large mass defect, but this defect can be explained by several sources. The theoretical mass does not consider hexon deamidation. As shown in the RP-HPLC/MS data, the observed hexon in the “impurity” and “main” peaks likely has several sites of deamidation present. If the monomeric hexon has on average 3 deamidations present on the capsid, the theoretical mass for the average trimer would be 9 Da higher than the theoretical mass listed in Table 3. Furthermore, deconvolution of spectra from native SEC-MS is challenging due to a limited number of charge states, higher m/z measurements, and poorer desolvation, often resulting in larger than expected masses. Given challenges in characterizing native spectra, and the fact that we know there is significant deamidation present on the hexon proteins in the 12-weeks accelerated stability sample, we feel that the observed mass is indeed reflective of hexon trimer.

### Linking Capsid Deamidation to an Acidic Shift in the AEX-HPLC Profile

**FIGURE 6:**
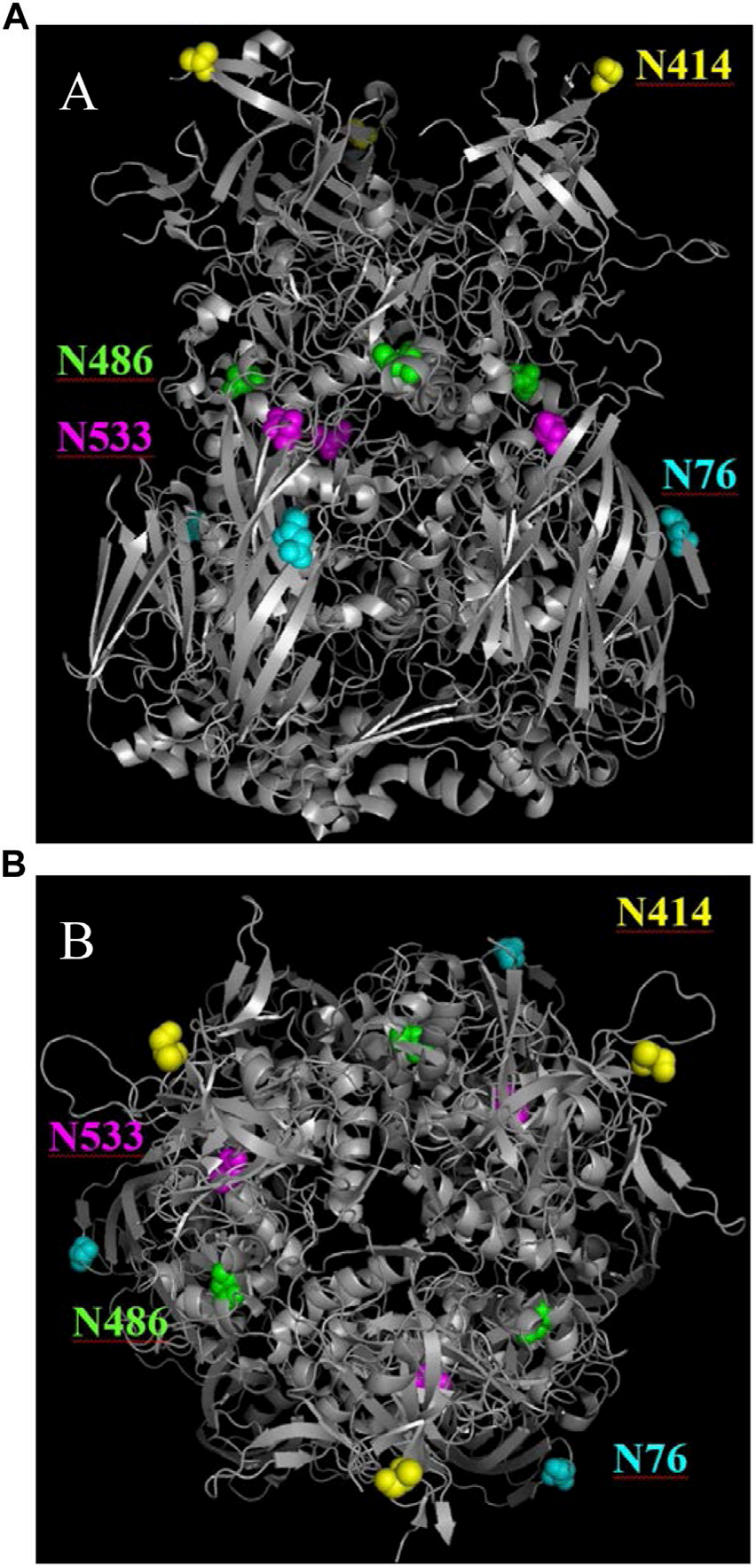
Ribbon diagrams of the hexon AdC68 crystal structure (2obe.pdb) with selected asparagine residues highlighted. **(A)** demonstrates that N414 is on the surface and N76 is buried within the trimer. **(B)** demonstrates that N486 and N533 are buried within the hexon.

As the change in purity and particle titer have been accounted for, the cause of the relative retention time shift was then explored. In previous work on human adenovirus (Blanche et al., 2001), a similar shift was observed and was isolated to the deamidation of four accessible hexon asparagine-glycine (NG) residue pairs. Such NG pairs are known hotspots for deamidation (Geiger and Clarke, 1987). The recombinant AdC68 constructs studied in the current manuscript had 3 such NG pairs in its hexon primary sequence: N414, N486 & N533, two of which align with the human adenovirus structure. Instead of focusing the search on just these residues, MS/MS data were evaluated in a data dependent manner to see if other sites of modifications were present and changing. Interestingly, N76 which has an asparagine-threonine (NT) pair, showed a deamidation propensity like N414, albeit at a slightly slower rate. The deamidation levels of N76, N414, N486 and N533 are plotted in Figure 3A. Overall, the high level of hexon deamidation aligns with the hypothesis of deamidation from the RP-HPLC/MS data at the intact capsid protein level. Furthermore, N414 deamidation was contrasted with the relative retention time, and the curves showed good correlation with a single exponential equation indicating a single kinetic process and similar rate constants (Figure 3B). N414 is at the external surface of the hexon trimer (Figure 6) and could be directly responsible for the acidic shift in the surface charge observed by AEX-HPLC.

To confirm this hypothesis, site-directed mutagenesis of N414 to D414 was performed, which mimics 100% deamidation at the N414 site. As expected, when the N414D mutant was run by AEX-HPLC, alongside the accelerated stability samples, the relative retention time was in line with the expected result based on the single exponential equation (Figure 5). While the relative retention time shift was explained by deamidation at N414, this mutation did not impact infectivity.

While deamidation at N76 is not unexpected based on the NT amino acid motif, structural analysis (Figure 6) shows that the N76 residue exists at the outer surface of the hexon trimer in a region that is buried on the viral particle surface (Reddy, 2017; Matteson et al., 2018). As such, we wanted to ensure that the deamidation was not observed only on the “impurity” fraction, which represents free hexon trimer. Therefore, we digested both the unfractionated 0-weeks and 12-weeks accelerated stability samples in addition to the “impurity” and “main” fractions and assessed the level of deamidation for both N76 and N414 (Figure 4). There is significant evidence that both N76 and N414 deamidation occurs on the “main” peak, which represents the assembled adenovirus capsid, as evidenced by the increase in deamidation compared to the 0-weeks sample. Furthermore, N414 was at roughly equivalent levels across all samples. While N76 deamidation was moderately more prominent in the “impurity” fraction compared to the “main” fraction, the abundance of deamidation in both fractions were still significantly larger than the 0-weeks sample. Thus, while moderate N76 deamidation may occur after particle degradation, there is still evidence that significant N76 deamidation occurs on assembled adenovirus capsid.

Site-directed mutagenesis of N76 to D76, as a mimic for the 100% deamidated residue was performed to investigate the impact of N76 deamidation on the relative retention time shift and infectivity. However, we were unable to form assembled particles after making this mutation. Collectively, evidence of deamidation at N76 at higher rates in the “impurity” peak and the inability to form capsids after site directed mutagenesis of N76 to D76, suggests that this modification may be deleterious to assembled capsids. Whatever the mechanism for its alteration, deamidation of N76 seems to be detrimental to the structural integrity of the viral capsid and might be a site of interest in future molecular designs to stabilize the product for long-term storage.

## Conclusion

At a high level, the manuscript overviews a critical method to support the analytical control strategy for adenovirus-based therapeutics. The analytical AEX-HPLC provides information on viral particle titer, particle purity, and surface charge. During development, it was determined that accelerated stability conditions impacted all reportable attributes in the AEX-HPLC assay and also resulted in a decrease in infectivity. While changes in purity and titer may account for some of the infectivity loss, there appears to be an additional mechanism driving infectivity loss. Deamidation accounts for the change in surface charge, but N414 deamidation did not impact infectivity. A new site of deamidation was identified and preliminary data suggests that this site may be deleterious to the capsid. A full assessment of the impact of N76 deamidation on infectivity would be an enlightening follow up manuscript. The current manuscript establishes AEX-HPLC as a stability-indicating method, bolsters the understanding of attributes that underpin changes in the AEX-HPLC profile, and establishes the AEX-HPLC approach as a platform method that can be applied to multiple candidates to accelerate product development.

## Data Availability

The datasets presented in this article are not readily available because Raw data may be deemed proprietary by legal. Requests to access the datasets should be directed to lawrence.thompson@pfizer.com.
