# Supplementary material for: Identification of Recombinant Chimpanzee Adenovirus C68 Degradation Products Detected by AEX-HPLC
Source: Front Bioeng Biotechnol. 2022 Apr 5;10:753481. doi: 10.3389/fbioe.2022.753481 (PMC9017566; doi:10.3389/fbioe.2022.753481)

**Supplemental Figure 1**: AEX-HPLC and RP-HPLC profiles of the AdC68 #2 12-week accelerated stability sample. The chromatographic profile of the unfractionated 12-week stability sample is shown in **A**. After enrichment of the “Impurity” and “Main” peaks by the preparative AEX-HPLC assay, efficient enrichment was demonstrated of the “Impurity” (**B**) and “Main” (**C**) peaks by the analytical AEX-HPLC method. Analyses of fractionated “Impurity” and “Main” peaks by RP-HPLC are shown in **D** and **E**, respectively.

**Supplemental Figure 2**: Deamidation of selected hexon asparagine residues during the accelerated stability time course. Panels **A** shows percent deamidation determined for N76, 414, 486 and 533 in AdC68 #2. Panel **B** shows percent deamidation for N414 and the AEX-HPLC relative retention time shift both fit to single exponential equations, the calculated rate constants and the associated error for AdC68 #2.

**Supplemental Figure 1**: AEX-HPLC and RP-HPLC profiles of the AdC68 #2 12-week accelerated stability sample. The chromatographic profile of the unfractionated 12-week stability sample is shown in A. After enrichment of the “Impurity” and “Main” peaks by the preparative AEX-HPLC assay, efficient enrichment was demonstrated of the “Impurity” (B) and “Main” (C) peaks by the analytical AEX-HPLC method. Analysis Analyses of fractionated “Impurity” and “Main” peaks by RP-HPLC is are shown in D and E, respectively.

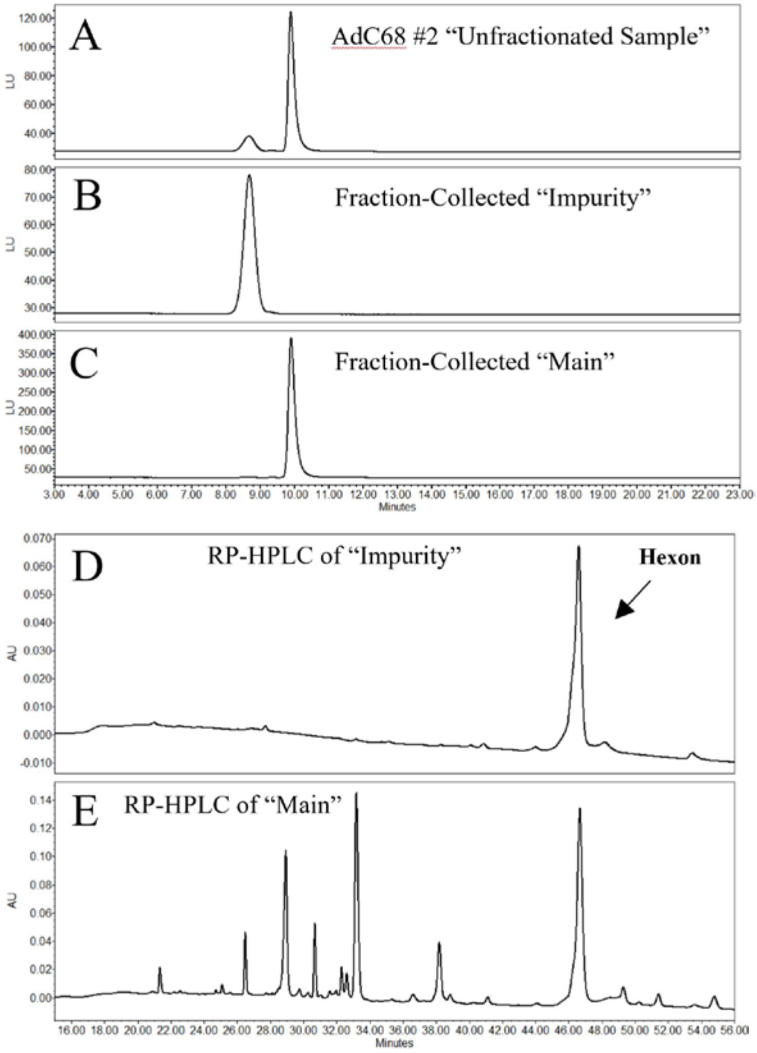

**Supplemental Figure 2**: Deamidation of selected hexon asparagine residues during the accelerated stability time course. Panels **A** shows percent deamidation determined for N76, 414, 486 and 533 in AdC68 #2. Panel **B** shows percent deamidation for N414 and the AEX-HPLC relative retention time shift both fit to single exponential equations, the calculated rate constants and the associated error for AdC68 #2.

A

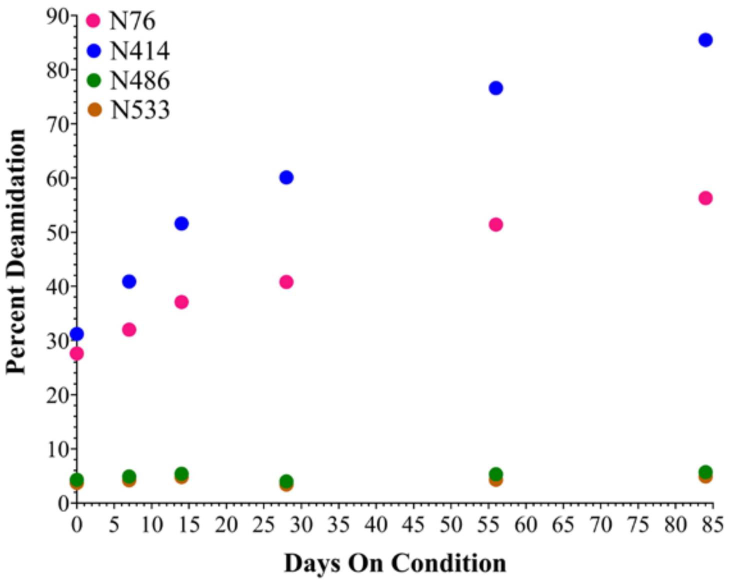


B

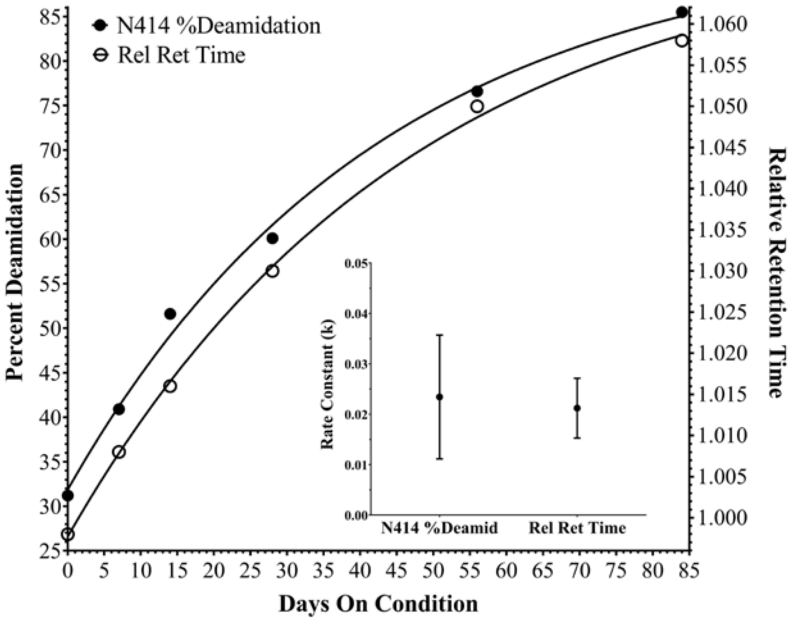

Supplement: Supplementary file 1 [file DataSheet1.pdf]
